# Conformation and Structure of Hydroxyethyl Cellulose Ether with a Wide Range of Average Molar Masses in Aqueous Solutions

**DOI:** 10.3390/polym14214532

**Published:** 2022-10-26

**Authors:** Misato Yoshida, Hiroki Iwase, Toshiyuki Shikata

**Affiliations:** 1 Division of Natural Resources and Eco-Materials, Graduate School of Agriculture, Tokyo University of Agriculture and Technology, 3-5-8, Saiwai-cho, Fuchu, Tokyo 183-8509, Japan; 2 Cellulose Research Unit, Tokyo University of Agriculture and Technology, 3-5-8, Saiwai-cho, Fuchu, Tokyo 183-8509, Japan; 3 Neutron Science and Technology Center, Comprehensive Research Organization for Science and Society (CROSS), 162-1 Shirakata, Tokai, Naka 319-1106, Ibaraki, Japan

**Keywords:** hydroxyethyl cellulose, small-to-wide-angle neutron scattering, small-to-wide-angle X-ray scattering, static light scattering, dynamic light scattering, weight average molar mass, radius of gyration, translational diffusion coefficient, rotational diffusion coefficient

## Abstract

The solution properties of a water-soluble chemically modified cellulose ether, hydroxyethyl cellulose (HeC), were examined using static light scattering (SLS), dynamic light scattering (DLS), small-to-wide-angle neutron scattering (S-WANS), small-to-wide-angle X-ray scattering (S-WAXS) and viscometric techniques at 25 °C. The examined HeC samples had average molar substitution numbers ranging from 2.36 to 2.41 and weight average molar masses (*M*_w_) that fell within a wide range from 87 to 1500 kg mol^−1^. Although the relationship between the determined radius of gyration (*R*_g_) and *M*_w_ was described as *R*_g_ ∝ *M*_w_^~0.6^, as is observed usually in flexible polymer solutions in good solvents, the observed scattering vector (*q*) dependencies of excess Rayleigh ratios were well interpreted using a rigid rod particle model, even in high-*M*_w_ samples. Moreover, the ratios of the formed particle length (*L*) evaluated assuming the model for rigid rods to the determined *R*_g_ showed the relationship *LR*_g_^−1^ ~ 3.5 irrespective of *M*_w_ and were close to those theoretically predicted for rigid rod particle systems, i.e., *LR*_g_^−1^ = $\sqrt{12}$. The observed SLS behavior suggested that HeC molecules behave just like rigid rods in aqueous solution. As the *L* values were not simply proportional to the average molecular contour length calculated from the *M*_w_, the chain conformation or structure of the formed particles by HeC molecules in aqueous solution changed with increasing *M*_w_. The *q* dependencies of excess scattering intensities observed using the S-WANS and S-WAXS experiments demonstrated that HeC molecules with *M*_w_ less than 200 kg mol^−1^ have a diameter of ~1.4 nm and possess an extended rigid rod-like local structure, the size of which increases gradually with increasing *M*_w_. The observed *M*_w_ dependencies of the translational and rotational diffusion coefficients and the intrinsic viscosity of the particle suspensions strongly support the idea that the HeC molecules behave as rigid rod particles irrespective of their *M*_w_.

## 1. Introduction

Hydroxyethyl cellulose (HeC) is a water-soluble chemically modified cellulose ether that is derived from cellulose by additional reaction with ethylene oxide by many manufacturers at present [1,2,3]. As HeC is obtained from an abundant and eco-friendly natural product, cellulose, it has been produced by many chemical manufacturers and is used in many practical applications in daily life; one such use is as an effective rheology modifying agent [4,5,6,7,8]. The fascinating rheological behavior of aqueous HeC solutions, including their shear and extensional behavior, has attracted much attention from rheologists in recent years [9,10]. The water solubility of HeC samples is controllable by the molar substitution number (*MS*) of the hydroxyethyl groups of each constituent glucose ring; most commercial water-soluble HeC samples have *MS* numbers between 1.3 and 3.4, and HeC samples maintain rather high water solubility over a wide temperature range irrespective of the weight average molar mass (*M*_w_) [11]. Other water-soluble chemically modified cellulose ethers such as methyl cellulose (MC) and hydroxypropylmethyl cellulose (HpMC) show a characteristic temperature dependence of water solubility; MC and HpMC are soluble in water only at temperatures lower than, e.g., 40 °C and show rather sharp clouding behavior and sometimes gelation above this temperature, depending on the *MS* numbers of the hydroxypropyl groups and the degree of substitution (*DS*) by methyl groups in each glucose unit [12]. The mechanisms through which the clouding and gelation phenomena observed in aqueous MC solutions occur have been investigated in detail [13,14]. The characteristic clouding behavior is understood to be a steep dehydration phenomenon that occurs with increasing temperature and in which the molecules fail to achieve the critical hydration state necessary to maintain water solubility. In the case of water-soluble HeC samples, the temperature dependence of the hydration number (*n*_H_) per glucose ring is much weaker than that of HpMC and MC samples, and *n*_H_ does not fall to the critical value at which the HeC clouds or gels in water, even at temperatures higher than 70 °C [15]. Thus, HeC samples possess stable fluidity even at higher temperatures without showing clouding and gelation phenomena; thus, they are useful in applications that require stable solubility and fluidity and provide the desired rheological behaviors in aqueous solutions over wide temperature ranges from 0 to 90 °C.

We recently reported that MC samples with a wide range of *M*_w_ possess a highly elongated rigid rod-like conformation and structure in aqueous solution at room temperature (25 °C) and that these MC samples maintain high solubility [16,17]. As many macromolecular scientists believe that water-soluble cellulose derivatives of sufficiently high *M*_w_ behave as semiflexible polymer chains with persistence lengths of, e.g., ~5 nm and act as flexible chains in aqueous solutions [18,19], the rigid rod-like structure found in MC samples over a wide *M*_w_ range is rather curious. Many investigators might ask the simple question “Do HeC samples that exhibit stable water solubility without showing clouding behavior in aqueous solutions also have conformations and structures identical to the rigid rod-like particles found in MC samples?” To obtain a clear answer to this question, we decided to investigate the conformation and structure in aqueous solution at room temperature (25 °C) of HeC samples with an *MS* value of ~2.4 and a wide range of *M*_w_ values ranging from less than 10^2^ kg mol^−1^ to greater than 10^3^ kg mol^−1^ using scattering techniques such as static light scattering (SLS), dynamic light scattering (DLS), small-to-wide-angle neutron scattering (S-WANS) and small-to-wide-angle X-ray scattering (S-WAXS) techniques and viscometric measurements. As the chemical modifications, *MS* values and *M*_w_ values of the HeC samples examined in this study do not differ from those of many commercially available HeC samples, the information obtained on the conformation and structure of the HeC samples in this work provides basic fundamental knowledge related to many types of applications of HeC in aqueous solution.

## 2. Experimental

### 2.1. Materials

The five HeC samples investigated in this study were supplied by Daicel Corporation (Tokyo; Japan), and were used without any further purification. The molar substitution numbers, *MS*, for each HeC sample were determined by use of the so-called hydrogen iodide decomposition reaction method, and the weight average molar masses were determined using SLS techniques. The determined *MS* values were somewhat dependent on the sample species and ranged from 2.36 to 2.41, as summarized in Table 1. The samples were coded using numbers related to their *M*_w_ (in kg mol^−1^); for example, the HeC sample with *M*_w_ = 87 kg mol^−1^ was coded “HeC87”. For each HeC sample, the polydispersity index, *M*_w_*M*_n_^−1^, in which *M*_n_ indicates the number average molar mass, was roughly determined using size-exclusion chromatography. The determined *M*_w_*M*_n_^−1^ values of all samples were greater than 2, as summarized in Table 1.

Deuterium oxide (D_2_O, deuterium content > 99.8%) was purchased from Eurisotope (Saint-Aubin; France) and used as a solvent in the S-WANS experiments. Highly deionized water with a specific electrical resistance greater than 18 MΩ cm obtained using a Direct-Q UV 3, Millipore (Darmstadt; Germany) was used as a solvent in the preparation of aqueous solutions of HeC used in experiments other than the S-WANS experiments.

HeC samples at several concentrations (*c*), each of which was lower than the reciprocal of the intrinsic viscosity ([*η*]^−1^) resulting from viscometric measurements, were used in the SLS and DLS experiments. The *c* values of the HeC87 samples in D_2_O used in the S-WANS measurements were *c* = 5.0 × 10^−3^ and 1.0 × 10^−2^ g mL^−1^. The *c* values in prepared solutions of the samples used in the S-WAXS experiments were *c* = 2.5 × 10^−3^, 5.0 × 10^−3^ and 1.0 × 10^−2^ for HeC87, *c* = 1.0 × 10^−3^ for HeC1000 and *c* = 8.0 × 10^−4^ g mL^−1^ for HeC1500.

### 2.2. Methods

SLS and DLS measurements were conducted using an extensively modified DLS7000 originally manufactured by Otsuka Electronics Co., Ltd. (Osaka, Japan). A single-frequency Sapphire laser, SF488-100, Coherent, Inc. (Santa Clara, CA, USA), at a wavelength of *λ* = 488.0 nm, was equipped with the modified DLS7000 as a light source. As a light detector, a photomultiplier, R9880U-01, Hamamatsu Photonics K. K. (Hamamatsu, Japan), was installed. To record intensity fluctuations in scattered light as a function of time, an LSI correlator, LS Instrument AG (Fribourg, Swiss), was used. An autocorrelation function of the scattered light intensity was precisely calculated by the correlator. A Pyrex glass tube which possesses an inner diameter of 19.0 mm and a thickness of 0.5 mm was used as a measuring cell. The measuring temperature for both SLS and DLS measurements was 25.0 °C. The scattering angle (*θ*) was varied from 30° to 150° in 10° increments. The magnitudes of the scattering vectors covered from 9.78 × 10^−3^ to 3.65 × 10^−2^ nm^−1^. Toluene was employed as the standard material for scattered light intensity in the SLS experiments. The refractive index increment (∂*n*/∂*c*) of the HeC in aqueous solution was determined to be 0.139 mL g^−1^ by use of an Abbemat MW multiwavelength refractometer, Anton Paar (Graz, Austria), at a wavelength of *λ* = 486 nm. This value was used to determine the *M*_W_ values for each HeC sample from the SLS data. ∂*n*/∂*c* = 0.138 mL g^−1^ was also determined at a different wavelength of *λ* = 589 nm^−1^, and the *λ* dependence of ∂*n*/∂*c* of the HeC samples was weak.

S-WANS experiments were conducted at the Materials and Life Science Experimental Facility (MLF) at the Japan Proton Accelerator Research Complex, J-PARC (Tokai, Japan), using a small-angle neutron scattering facility (TAIKAN) [20], which is installed on a beamline BL15. The range of the scattering vector, *q*, used in the S-WANS experiments covered from 9.0 × 10^−2^ to 1.0 × 10^2^ nm^−1^. As sample cells for S-WANS experiments, square-type quartz cuvettes with a neutron beam path length of 4.0 mm were used. The standard exposure time was 2 h for each sample solution. S-WANS measurements were conducted at 25 °C. The obtained scattering data were converted to absolute values by use of a standard “glassy carbon” data. Absolute scattering value for the glassy carbon had been precisely determined.

S-WAXS measurements were conducted using a small-to-wide-angle X-ray scattering analyzer, SAXSpace, Anton Paar(Graz, Austria), at 25 °C. The scattering vector, *q*, in the S-WAXS measurements ranged from 1.3 × 10^−1^ to 1.9 × 10^1^ nm^−1^. Quartz capillaries with outer diameter and thickness of 1.0 nm and 10 μm, respectively, were used as sample cells. The X-ray exposure time was 8 h for aqueous HeC solutions at *c* ≤ 2.5 × 10^−3^ g mL^−1^ and 30 min for aqueous HeC87 solutions at *c* = 5.0 × 10^−3^ and 1.0 × 10^−2^ g mL^−1^.

The intrinsic viscosities, [*η*], of HeC samples in aqueous solutions were determined at 25.0 °C with a Ubbelohde-type capillary viscometer.

## 3. Results and Discussion

### 3.1. SLS Behaviors

**Figure 1:**
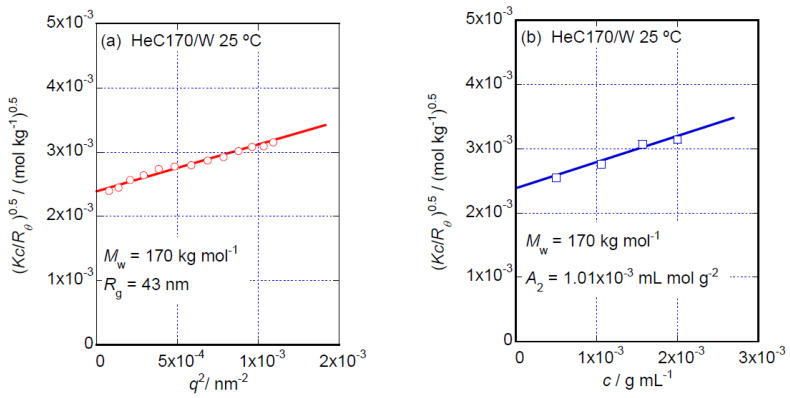
*q*^2^ dependencies of the ${\left(\sqrt{Kc{\left(R_{\theta }\right)}^{-1}}\right)}_{c=0}$ data (**a**) and *c* dependencies of the ${\left(\sqrt{Kc{\left(R_{\theta }\right)}^{-1}}\right)}_{q=0}$ data (**b**) for aqueous solutions of the HeC170 sample at 25 °C.

Using values of *λ* = 488.0 nm for light course and ∂*n*/∂*c* = 0.139 mL g^−1^ for the examined HeC in aqueous solution, the apparatus constant (*K*) of the system was determined to be *K* = 3.89 × 10^−7^ cm^2^ mol g^−2^. The excess Rayleigh ratios, *R_θ_*, compared to the standard liquid, toluene, were determined at each scattering angle for all the aqueous HeC solutions examined. As one of the typical SLS experimental results, the square of the scattering vector, *q*^2^, and the dependencies of the ${\left(\sqrt{Kc{\left(R_{\theta }\right)}^{-1}}\right)}_{c=0}$(= $\underset{c\to 0}{\lim}\sqrt{Kc{\left(R_{\theta }\right)}^{-1}}$) data, so-called Berry plots [21], for the HeC170 sample are shown in Figure 1a. As the HeC samples possess rather broad molar mass distributions, Berry plots rather than Zimm plots [22] were used to analyze the data in this study. The intercept determined from the solid line in Figure 1a indicates *M*_w_ = 170 kg mol^−1^, and the initial slope of the line indicates a radius of gyration *R*_g_ = 43.0 nm for HeC170. The *c* dependencies of the ${\left(\sqrt{Kc{\left(R_{\theta }\right)}^{-1}}\right)}_{q=0}$(= $\underset{q\to 0}{\lim}\sqrt{Kc{\left(R_{\theta }\right)}^{-1}}$) data for the same HeC170 sample are shown in Figure 1b. The intercept determined from the solid line in Figure 1b yields the same *M*_w_ value as that obtained using the solid line in Figure 1a, and the initial slope of the line indicates a second virial coefficient of *A*_2_ = 1.0 × 10^−3^ mL mol g^−2^ for HeC170. Using essentially the same procedures as described above, the values of *M*_w_, *R*_g_ and *A*_2_ were determined for each HeC sample examined in this study. Table 1 summarizes these values.

**Figure 2:**
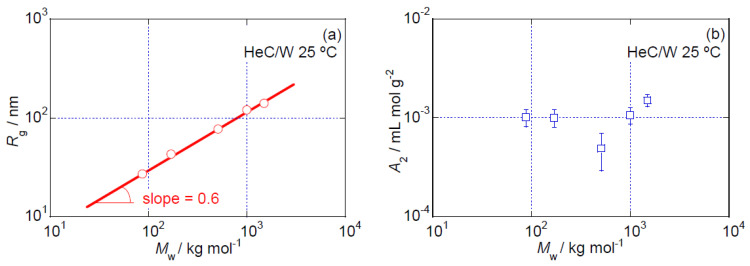
*M*_w_ dependencies of *R*_g_ (**a**) and *A*_2_ (**b**) for HeC samples in aqueous solution at 25 °C.

The dependencies of *R*_g_ and *A*_2_ on the *M*_w_ values of the HeC samples are shown in Figure 2a,b, respectively. Based on the slope of the solid line in Figure 2a, which is ~0.6, the relationship *R*_g_ ∝ *M*_w_^0.6^ is approximately obtained. This *M*_w_ exponent is close to the values that are usually reported for flexible polymer chains dissolved in moderately good solvents [23]. However, we cannot simply conclude that the HeC samples behave as flexible polymer chains in aqueous solution, because the *q* dependence of the (*R_θ_* (*Kc*)^−1^)*_c_*_=0_ obtained in the SLS experiments and that of the S-WANS and S-WAXS data, which are discussed in detail in later sections, do not show flexible polymer chain behavior. The value of *A*_2_ indicates the strength of the interparticle interaction between two solute molecules. The decrease in the *A*_2_ value observed in Figure 2b up to the *M*_w_ value of ~500 kg mol^−1^ indicates that there is a decrease in the repulsive interaction between two HeC molecules with increasing *M*_w_ that leads to an increase in attractive interactions between HeC molecules with increasing *M*_w_. After reaching its minimum, *A*_2_ displays a steep increase in its *M*_w_ dependence, as is clearly seen in Figure 2b; this is related to an increase in repulsive interactions between HeC molecules with increasing *M*_w_. The observed complicated *M*_w_ dependence of *A*_2_ in the HeC samples appears to be caused by conformational and structural changes induced by alterations in the intramolecular interactions between constituent segments of HeC molecules with increasing *M*_w_.

As the *A*_2_ values of most flexible and semiflexible polymer chains that maintain constant persistence lengths irrespective of *M*_w_ when dissolved in solutions show monotonous weak decreasing behavior with increasing *M*_w_ [23,24], the *M*_w_ dependence of *A*_2_ observed in Figure 2b suggests a clear difference between the HeC molecules analyzed in this study and the usual flexible and semiflexible polymer chains from the viewpoint of intermolecular interactions. In the case of aqueous solutions of another water-soluble chemically modified cellulose ether, methyl cellulose, MC, the observed *A*_2_ decreases rather drastically with increasing *M*_w_ and does not show a subsequent steep increase up to an *M*_w_ value of ~1.0 × 10^3^ kg mol^−1^, as observed in Figure 2b [17]. Thus, it is likely that the structural change that occurs in the aqueous HeC samples is not identical to that in the aqueous MC samples [17].

**Figure 3:**
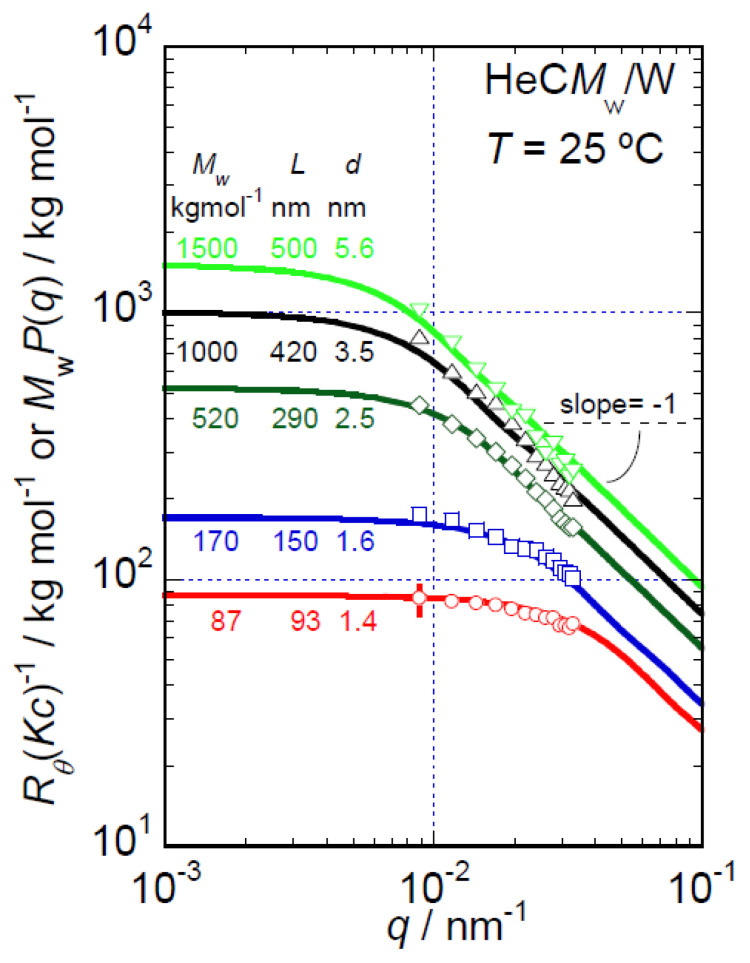
*q* dependencies of the *R_θ_*(*Kc*)^−1^*_c_*_=0_ data and fitted *M*_w_*P*(*q*) curves for HeC samples in aqueous solution at 25 °C.

The *q* dependencies of *R_θ_*(*Kc*)^−1^*_c_*_=0_ data for all HeC samples are shown in Figure 3 on a double-logarithmic scale. For HeC samples with *M*_w_ values lower than 200 kg mol^−1^, the *R_θ_*(*Kc*)^−1^*_c_*_=0_ data show weak *q* dependencies, for which suitable form factors, *P*(*q*), cannot be obtained precisely from the SLS data alone. However, the HeC samples with lower *M*_w_ values demonstrated obvious proportionality to *q*^−1^ in their excess scattering intensities, Δ*I*(*q*)*c*^−1^, in the data obtained in the S-WANS and S-WAXS experiments covering a *q* range higher than that of the SLS experiments, as discussed in detail in a later section. In a high *q* range of the SLS data, the HeC samples with *M*_w_ values higher than 500 kg mol^−1^ clearly show the relationship *R_θ_*(*Kc*)^−1^*_c_*_=0_ ∝ *q*^−1^, as seen in Figure 3. As the relationship *R_θ_*(*Kc*)^−1^*_c_*_=0_ (or Δ*I*(*q*)*c*^−1^) ∝ *q*^−1^ indicates characteristic behavior of the form factors, *P*(*q*), of rigid rod particles in the *q* range higher than the reciprocal of rod particle length (*L*) [25,26] and *R_θ_*(*Kc*)^−1^*_c_*_=0_ theoretically corresponds to *M*_w_*P*(*q*), it is possible that the *R_θ_*(*Kc*)^−1^*_c_*_=0_ data for all the HeC samples in this study are describable based on the form factors of rigid rod particles that possess different *L* values that depend on the *M*_w_ of the sample.

The solid lines shown in Figure 3 represent *M*_w_*P*(*q*) curves resulting from curve fitting to the *R*_θ_(*Kc*)^−1^*_c_*_=0_ data for each HeC sample assuming rigid rod particle form factors. In the curve fitting procedure, the open source software SasView [27] was employed to calculate rigid rod form factors [26], *P*(*q*). Varying *L* values, *M*_w_*P*(*q*) curves were fitted to the *R_θ_*(*Kc*)^−1^*_c_*_=0_ data to permit identification of the most adequate *L* values for each HeC sample. The *q* dependence of the *P*(*q*) curves in the *q* range covered by the SLS experiments (*q* < 4 × 10^−2^ nm^−1^) was not sensitive to the diameter (*d*) of the assumed rigid rod particles when *d* was set at values smaller than, e.g., 10 nm. The values of *L* (and *d*) determined as the most adequate average values based on fitting of the data are shown in Figure 3. As the observed agreement between the *M*_w_*P*(*q*) curves and the *R_θ_*(*Kc*)^−1^*_c_*_=0_ data is reasonable for HeC samples with *M*_w_ greater than 500 kg mol^−1^, the rigid rod particle form factors appear to be suitable for describing the SLS behavior of HeC samples in aqueous solutions. The *R_θ_*(*Kc*)^−1^*_c_*_=0_ data for the HeC samples with *M*_w_ values lower than 100 kg mol^−1^ display quite weak *q* dependence in the *q* range covered by the SLS experiments for the determination of *M*_w_*P*(*q*) curves precisely. Then, the combination of the *q* dependencies of the *R_θ_*(*Kc*)^−1^*_c_*_=0_ data and the excess scattering intensity data, Δ*I*(*q*)*c*^−1^, resulting from the S-WANS and S-WAXS experiments will be quite useful to determine *M*_w_*P*(*q*) curves for the low-*M*_w_ HeC samples as discussed in detail in the next section.

**Figure 4:**
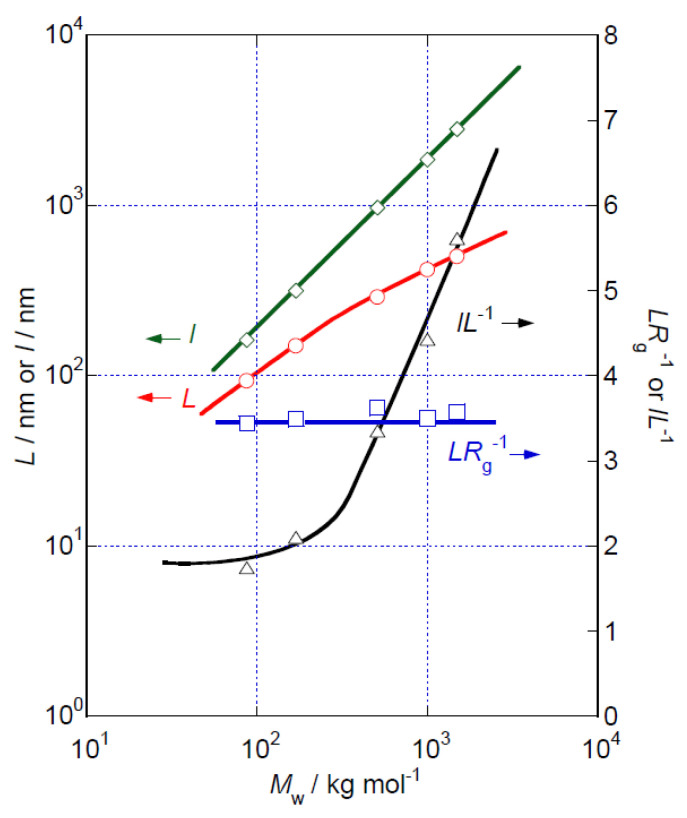
*M*_w_ dependencies of *L*, *LR*_g_^−1^ and *lL*^−1^ for HeC samples in aqueous solution at 25 °C. Lines are drawn in this figure as a guide for the eye.

Figure 4 shows the *M*_w_ dependencies of *L* and *LR*_g_^−1^ determined for each HeC sample. Although the value of *L* increases monotonically with increasing *M*_w_, the *L* value is not proportional to the *M*_w_ value, and the ratio *LR*_g_^−1^ appears to remain at a constant value close to ~3.5 irrespective of *M*_w_. As the relationship found in this study is close to the simple relationship *L*^2^ = 12*R*_g_^2^, i.e., *L*(*R*_g_)^−1^ = $\sqrt{12}$ ~ 3.46, which holds in suspensions of rigid rod particles, the results might be interpreted as strongly supporting a rigid rod structure of the particles formed by HeC molecules in aqueous solution. However, the HeC molecules cannot have simply elongated straight conformations or systematic helical conformations because the *L* of the formed particles is not simply proportional to *M*_w__,_ as described above. The average contour lengths (*l*) of the HeC molecules in each HeC sample can be calculated assuming the repeating length of the glucose unit to be 0.5 nm [28], and in this way a holding number defined as *lL*^−1^, which indicates the average molecular chain number per cross section of the rod particles formed by the HeC molecules, can be calculated, considering that an essential characteristic of cellulose molecules that form the framework of HeC is that they prefer to assume a straight elongated conformation. The *M*_w_ dependence of the *lL*^−1^ value for HeC samples is also shown in Figure 4. For HeC samples of *M*_w_ less than 20 kg mol^−1^, the *lL*^−1^ value is not far from two. This observation suggests that short HeC molecules have hairpin-like particle structures with *lL*^−1^ = 2 on average. The presence of a similar hairpin-like structure has already been demonstrated in aqueous solutions of MC and HpMC [16,17]. The observed change in the *lL*^−1^ value with increasing *M*_w_ in Figure 4 suggests alterations in the size and shape of the cross sections of HeC molecules in aqueous solution. However, the SLS data cannot provide useful information in the length scale shorter than 10 nm^−1^, which is related to the cross section of HeC molecules.

### 3.2. S-WANS and S-WAXS Behaviors

**Figure 5:**
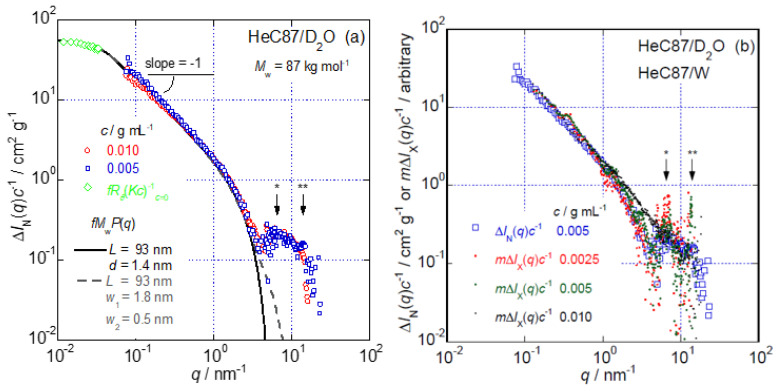
(**a**) *q* dependencies of the Δ*I*_N_(*q*)*c*^−1^ (S-WANS) data for D_2_O solutions of HeC87 at *c* = 0.005 and 0.010 g mL*^−^*^1^ at 25 °C; (**b**) *q* dependencies of the *m*Δ*I*_X_(*q*)*c*^−1^ (S-WAXS) data for aqueous HeC87 solutions at *c* = 0.0025, 0.0050 and 0.010 g mL^−1^ at 25 °C and that of Δ*I*_N_(*q*)*c*^−1^ data for the same HeC87 sample shown in (**a**). The SLS data obtained for the same system, *fR_θ_*(*Kc*)^−1^, and the two fit curves, *fM*_w_*P*(*q*), obtained assuming *f* = 6.5 × 10^−4^ cm^2^ g^−2^ mol, are also shown in (**a**). The numerical constant of *m* = 0.17 is used for all the *m*Δ*I*_X_(*q*)*c*^−1^ data in (**b**). The places of * and ** in the figures mean the positions of the broad interference-type peaks.

The *q* dependencies of concentration-reduced excess scattering intensities, Δ*I*_N_(*q*)*c*^−1^, determined via the S-WANS measurements for HeC87 at *c* = 0.005 and 0.010 g mL*^−^*^1^, are shown in Figure 5a. As the [*η*] value for HeC87 was determined to be 180 mL g^−1^, the Δ*I*_N_(*q*)*c*^−1^ data obtained at *c* = 0.005 g mL^−1^ should be more reliable than those obtained under the condition of the so-called isolated state for HeC molecules given by *c* < [*η*]^−1^. The difference between the Δ*I*_N_(*q*)*c*^−1^ data obtained at these concentrations is small, as seen in Figure 5a. Thus, the condition at *c* = 0.010 g mL^−1^ is not far from the isolated state. One can easily recognize the relationship Δ*I*(*q*)*c*^−1^ ∝ *q*^−1^ in the *q* range 0.1 nm^−1^ < *q* < 1.0 nm^−1^; such a relationship is characteristic of rigid rod or long columnar particles with *L* > 10 nm. A steeper decrease in the Δ*I*(*q*)*c*^−1^ data is observed over the *q* range greater than 2.0 nm^−1^; there, two broad interference-type peaks are clearly recognized at *q* ~ 6.5 and 14 nm^−1^, as seen in Figure 5a. As the presence of two similar interference-type peaks has also been observed in aqueous solutions of MC and HpMC, it is speculated that the local structures assumed by several water-soluble chemically modified cellulose ethers have common characteristics showing these peaks [16,17]. The observed peak at ~6.5 nm^−1^ corresponds to the periodic distance of ~(2π/6.5) 1.0 nm, close to the molecular size of cellobiose, the structural repeating unit of cellulose. Another periodic distance of 0.4 nm corresponding to the other peak at 14 nm^−1^, 2π/14 ~ 4.5 nm, is not far from the separation between facing cellulose molecules in the crystalline structure of cellulose II [16,17]. 

The solid line in Figure 5a shows the curve fit to the Δ*I*(*q*)*c*^−1^ data by SasView [27] assuming the rigid rod particle form factor, *P*(*q*), with *L* = 93 and *d* = 1.4 nm. Although the *L* value cannot be determined uniquely from the S-WANS data alone, the SLS data providing the value of *R*_g_ = 27 nm and the weak *q* dependence of *R_θ_*(*Kc*)^−1^*_c_*_=0_ data in the *q* range lower than 0.04 nm^−1^ were useful in determining the *L* and *d* values. The SLS data multiplied by the constant *f* = 6.5 × 10^−4^ cm^2^ g^−2^ mol, i.e., *fR_θ_*(*Kc*)^−1^*_c_*_=0_, are also plotted in Figure 5a. The value *f* = (6.5 ± 0.3) × 10^−4^ cm^2^ g^−2^ mol connects the S-WANS and SLS data quite smoothly using the rigid rod form factor, maintaining the characteristics of almost flat *P*(*q*) in the range of *q* < 0.04 nm^−1^ and *P*(*q*) ∝ *q*^−1^ in the range of 0.1 nm^−1^ ≤ *q* < 2.0 nm^−1^. The agreement between the solid fit curve, *fM*_w_*P*(*q*), with the identical *f* value to the *fR_θ_*(*Kc*)^−1^*_c_*_=0_ data and the experimental Δ*I*_N_(*q*)*c*^−1^ data appears reasonable except for the presence of the two interference-type peaks. The broken line in the same figure represents the fit curve calculated assuming a rectangular columnar particle with *L* = 93 nm, width *w*_1_ = 1.8 nm and thickness *w*_2_ = 0.5 nm. As the difference between the fit curves indicated by the solid and broken lines is inconspicuous, especially in the *q* range below 2.5 nm^−1^, we might conclude that these structural factors describe the local structure of HeC87 molecules rather reasonably. An elliptical rod particle [29] with structural parameters similar to those of the rectangular columnar particle would have a form factor, *P*(*q*), suitable for the Δ*I*_N_(*q*)*c*^−1^ data seen in Figure 5a.

The S-WAXS measurements covered a similar *q* range to the S-WANS measurements in this study. To confirm the validity of the data obtained using these two techniques, we compared the *q* dependencies of the Δ*I*_X_(*q*)*c*^−1^ data determined in S-WAXS measurements with those obtained from S-WANS measurements. As the Δ*I*_X_(*q*)*c*^−1^ data were not converted into absolute values in this study, the *m*Δ*I*_X_(*q*)*c*^−1^ data multiplied by a certain numerical constant, *m*, can be used for comparison. Figure 5b shows the *q* dependence of Δ*I*_N_(*q*)*c*^−1^ for the HeC87 sample obtained at *c* = 0.005 g mL^−1^ (the same data as seen in Figure 5a) and that of *m*Δ*I*_X_(*q*)*c*^−1^ at *c* = 0.0025, 0.0050 and 0.010 g mL^−1^ using the numerical constant *m* = 0.17. Although all the *c* values are rather low and the obtained Δ*I*_N_(*q*)*c*^−1^ and *m*Δ*I*_X_(*q*)*c*^−1^ data are poorly dispersed, the agreement between the two sets of data, with the exception of the *m*Δ*I*_X_(*q*)*c*^−1^ data at *c* = 0.010 g mL^−1^, is fairly good, especially in the *q* range 0.1 to 4.0 nm^−1^; within that range, the scattering data are proportional to *q*^−1^ up to *q* = 1.0 nm^−1^ and decrease more steeply above that *q* value. The data obtained at *c* = 0.010 g mL^−1^ clearly show substantially greater values than other data in the *q* range from 1.5 to 5.0 nm^−1^ and show the interference-type peaks less significantly than *m*Δ*I*_X_(*q*)*c*^−1^ data at other lower *c*. It appears that the *m*Δ*I*_X_(*q*)*c*^−1^ data obtained from the S-WAXS measurements demonstrate the presence of the two interference peaks at *q* = 6.5 and 14 nm^−1^ slightly more clearly than do the Δ*I*_N_(*q*)*c*^−1^ (S-WANS) data. Based on the observed fair agreement between the Δ*I*_N_(*q*)*c*^−1^ data and the *m*Δ*I*_X_(*q*)*c*^−1^ data in the *q* range 0.1 to 4.0 nm^−1^, we might conclude that both the S-WANS measurements and the S-WAXS measurements show the precise *q* dependence of scattering data for isolated HeC molecules under the condition of *c* ≤ [*η*]^−1^, as we expected.

**Figure 6:**
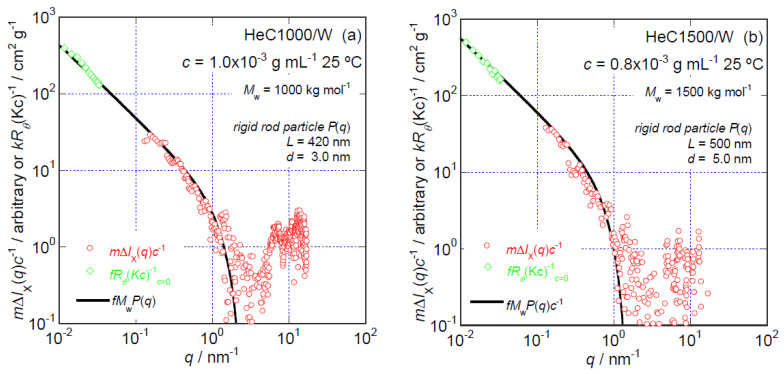
*q* dependencies of the *m*Δ*I*_X_(*q*)*c*^−1^ (S-WAXS) data obtained for HeC1000 at *c* = 1.0 × 10^−3^ g mL^−1^ (**a**) and those obtained for HeC1500 at *c* = 0.8 × 10^−3^ g mL^−1^ at 25 °C (**b**). The same numerical constants as those used to generate the fitted curves shown in Figure 5b, *f* = 6.5 × 10^−4^ cm^2^ g^−2^ mol and *m* = 0.17, were employed.

The *m*Δ*I*_X_(*q*)*c*^−1^ data for HeC1000 at *c* = 1.0 × 10^−3^ g mL^−1^ and those for HeC1500 at *c* = 0.8 × 10^−3^ g mL^−1^ are shown in Figure 6a,b, respectively. The *fR_θ_*(*Kc*)^−1^*_c_*_=0_ data for the HeC1000 and HeC1500 samples obtained from the SLS measurements and the *fM*_w_*P*(*q*) curves resulting from the rigid rod particle model are also included in Figure 6a,b. As the *f* and *m* values used in Figure 5b are also employed in Figure 6a,b, the *q* dependencies of the *m*Δ*I*_X_(*q*)*c*^−1^ data in these figures are directly comparable with the *fR_θ_*(*Kc*)^−1^*_c_*_=0_ data and the *fM*_w_*P*(*q*) curves quantitatively with respect to their magnitude. It is likely that the *q* range over which the relationship *m*Δ*I*_X_(*q*)*c*^−1^ ∝ *q*^−1^ is observed becomes narrower with increasing *M*_w_. In the case of HeC1000, the *m*Δ*I*_X_(*q*)*c*^−1^ data show a steeper decrease in magnitude with a negative exponent of *q*, obviously greater than unity, in the *q* range higher than ~0.8 nm^−1^, as seen in Figure 6a. A similar change in the *q* dependence of *m*Δ*I*_X_(*q*)*c*^−1^ data can also be recognized for HeC1500 in the *q* range higher than ~0.3 nm^−1^; this range is clearly lower than the *q* range observed for the solution of HeC1000(850), as seen in Figure 6b. This suggests that there is a change in the local particle structure formed by HeC molecules that is a function of *M*_w_. An increase in the diameter, *d*, of the rigid rod decreases the *q* range, showing the relationship *m*Δ*I*_X_(*q*)*c*^−1^ ∝ *q*^−1^. The solid line indicating *fM*_w_*P*(*q*), shown in Figure 6a, is the fit curve for the *m*Δ*I*_X_(*q*)*c*^−1^ data calculated using SasView [27] assuming the form factor of a rigid rod, *P*(*q*), with *L* = 420 nm and *d* = 3.0 nm. The agreement between the calculated curve and the data is reasonable in the *q* range below 1.0 nm^−1^. It is likely that the *m*Δ*I*_X_(*q*)*c*^−1^ data have a certain characteristic *q* dependence that is related to the local structure of the formed particles in the *q* range higher than 2.0 nm^−1^. However, the poor quality of the obtained data due to the low concentrations of HeC used in the experiments did not allow us to distinguish between a more local particle structure formed by HeC1000 molecules and other sophisticated particle models such as rectangular columnar and/or elliptical rod models.

HeC1500 molecules also appear to have rigid rod-like local structures, as clearly suggested by the dependence of the *m*Δ*I*_X_(*q*)*c*^−1^ data on *q* seen in Figure 6b. The solid line representing *fM*_w_*P*(*q*) in Figure 6b indicates the form factor, *P*(*q*), of a rigid rod with *L* = 500 nm and *d* = 5.0 nm as a function of *q*. The agreement between the fitted curve obtained using the rigid rod particle model and the *m*Δ*I*_X_(*q*)*c*^−1^ data is fair, especially in the *q* range lower than 1.0 nm^−1^. We did not consider more local structures of particles formed by HeC1500(900) in aqueous solution due to the poor quality of the data in the *q* range higher than 1 nm^−1^, as also seen in Figure 6b. However, despite the low quality of the data, two interference peaks at approximately 6.5 and 14 nm^−1^ can be discerned, as seen in Figure 6a,b.

The fair agreement between the fitted curves obtained assuming the form factors of rigid rods with diameter, *d*, varying from 1.4 to 5.0 nm with increasing *M*_w_ and the *m*Δ*I*_X_(*q*)*c*^−1^ data obtained for aqueous solutions of HeC samples, as seen in Figure 5 and Figure 6, strongly suggests that the particles formed by the HeC molecules can be described as having local structures and conformations that correspond to rigid rods with diameters that increase gradually with increasing *M*_w_.

### 3.3. Viscometric Behaviors

**Figure 7:**
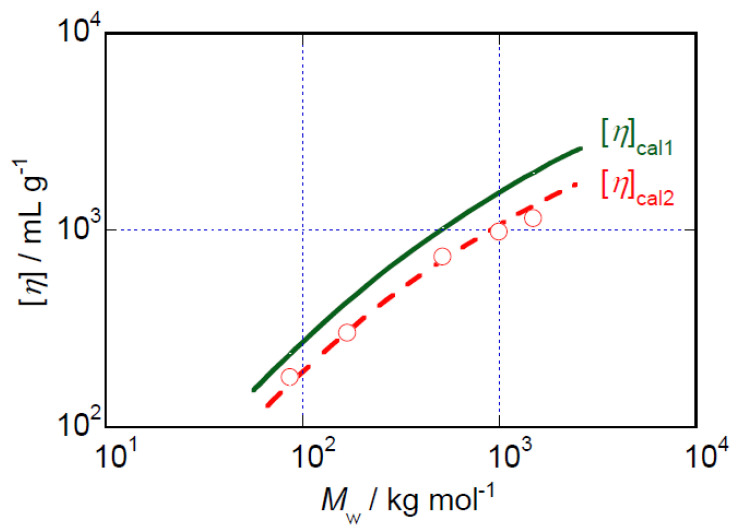
*M*_w_ dependence of [*η*] data for HeC samples in aqueous solution at 25 °C. The solid line labeled [*η*]_cal1_ in this figure shows the *M*_w_ dependence of [*η*]_cal_ assuming the first simple relationship, *L*_η_ = *L* and *d*_η_ = *d*, and the broken line labeled [*η*]_cal2_ represents the *M*_w_ dependence obtained assuming the second relationship, *L*_η_ = 0.87*L* and *d*_η_ = *d*.

The *M*_w_ dependence of the intrinsic viscosity, [*η*], of the HeC samples in aqueous solution is shown in Figure 7. Although the relationship [*η*] ∝ *M*_w_^~0.67^, which is usually observed for flexible polymer chain samples dissolved in good solvents [23], appears at a glance to hold, we would like to consider this relationship based on the idea of formation of rigid rod particles by the HeC samples. This consideration is based on the fact that all the scattering data obtained in the SLS and S-WANS and S-WAXS experiments can be fairly well explained using a form factor corresponding to that of rigid rod particles. Based on this consideration, we do not think that the lengths, *L*, of the formed particles are simply proportional to *M*_w_ and that the radii, *d*, of the formed rigid rod particles maintain a constant value irrespective of *M*_w_, despite the fact that this is a usually accepted idea.

According to theoretical calculations [30,31], the intrinsic viscosity of a suspension of rigid rod particles ([*η*]_cal_) with viscometric particle length (*L*_η_) and *d*_η_ is given as Equation (1).

(1) $${\left[\eta \right]}_{\mathrm{cal}}=\frac{2\pi N_{A}L_{\eta }{}^{3}}{45M_{w}\left\{\ln(L_{\eta }/d_{\eta })+C_{\eta }\right\}}$$

In Equation (1), *N*_A_ and *C*_η_ represent, respectively, Avogadro’s number and a numerical constant that shows the contribution of hydrodynamic interaction formulated using polynomials of the ratio *L*_η_/*d*_η_ [30]. As the first simple choice, we can select the relationship *L*_η_ = *L* and *d*_η_ = *d* to calculate [*η*]_cal_. It is known that *C*_η_ approaches a constant value of −0.93 when *L*_η_ is much longer than *d*_η_. We used this value in our calculations of [*η*]_cal_. The solid line representing [*η*]_cal1_ in Figure 7 shows the *M*_w_ dependence of [*η*]_cal_ resulting from the first simple choice. Although the agreement between the calculated [*η*]_cal1_ curve and the experimental data is not perfect, the *M*_w_ dependence of the [*η*] data is reproduced semiquantitatively. We then tested a second assumption, *L*_η_ = 0.87*L* and *d*_η_ = *d*; the curve obtained in that way is shown in Figure 7 as the broken curve labeled [*η*]_cal2_. The agreement between the [*η*]_cal2_ curve and the [*η*] data is much better than that between [*η*]_cal1_ and the [*η*] data. Changing the *d*_η_ value, e.g., using *d*_η_ = 0.87*d,* hardly affected the value of [*η*]_cal_. Consequently, we might conclude that the viscometric behavior of HeC samples in aqueous solution is reasonably described as corresponding to that of rigid rod particles with viscometric lengths slightly shorter than the structural lengths determined using scattering methods such as SLS, S-WANS and S-WAXS. Viscometric lengths that are shorter than structural lengths have also been reported for aqueous solutions of MC samples [17] and for *N*-methylpyrrolidone solutions of poly(vinylidene difluoride) [32].

### 3.4. Diffusional Behaviors 

**Figure 8:**
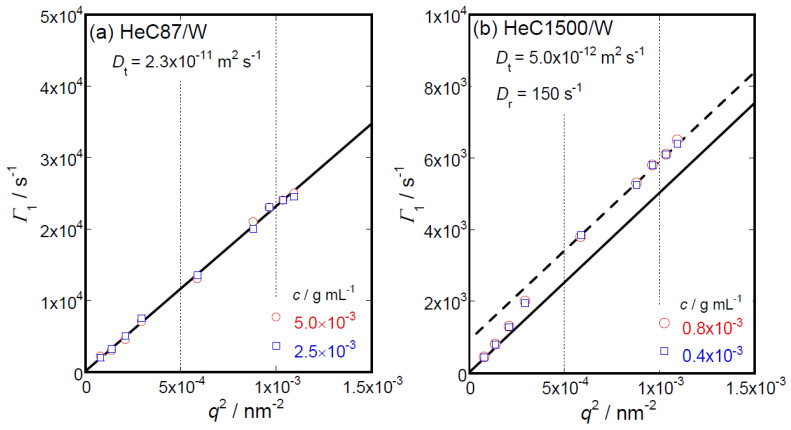
*q*^2^ dependencies of the *Γ*_1_ data for the shortest (HeC87, (**a**)) and the longest (HeC1500, (**b**)) samples. The broken line in (**b**) demonstrates the relationship *Γ*_1_ = 6*D*_r_ + *D*_t_ *q*^2^; thus, the *D*_r_ value can be evaluated from the intercept of the broken line at *q*^2^ = 0 nm^−2^.

The first cumulants, *Γ*_1_, calculated from the initial slopes of the obtained autocorrelation functions of the scattered light electric field provide translational diffusion coefficients, *D*_t_, of particles formed by solute molecules dissolved in sample solutions. As typical experimental results for HeC samples in aqueous solutions, Figure 8a,b show the *q*^2^ dependencies of *Γ*_1_ data for the shortest (HeC87) and the longest (HeC900) samples, respectively. The *Γ*_1_ data for the HeC87 sample seen in Figure 8a can be described as following a straight line with a constant slope over the *q*^2^ range covered in this study. In this case, the value of *D*_t_ can be simply evaluated as the constant slope of 2.3 × 10^−11^ m^2^ s^−1^. However, the *Γ*_1_ data for the HeC1500 sample shown in Figure 8b are not simply proportional to *q*^2^; instead, they show a sigmoidal shape dependence. For *Γ*_1_ data without linear *q*^2^ dependence, the *D*_t_ value can be obtained from the initial slope of the line conforming to the *Γ*_1_ data using the equation *D*_t_ = lim*_q_*^2^_→0_ *Γ*_1_*q*^−2^. In the case of large particles such as HeC1500, the *Γ*_1_ data reflect the contribution of rotational diffusion, especially in the high-*q*^2^ region. When the *Γ*_1_ data possess another *Γ*_1_ ∝ *q*^2^ relationship for which the proportional coefficient is identical to *D*_t_ for the high-*q*^2^ region, the *q*_2_ dependence of the *Γ*_1_ data can be approximately described as *Γ*_1_ = 6*D*_r_ + *D*_t_ *q*^2^ [17,33,34]. The rotational diffusion coefficient, *D*_r_, can then be evaluated from the intercepts of the straight lines, which follow the *Γ*_1_ data in the high-*q*^2^ range and are extrapolated to *q*^2^ = 0. This is shown in Figure 8b for the HeC1500 sample. However, in this study, the values of *D*_r_ were obtained only for the three high-*M*_w_ HeC samples. Although depolarized dynamic light scattering (DDLS) techniques [33] are better methods for determining *D*_r_ values for solute particles and we, of course, performed some DDLS measurements, scattering intensity data sufficient to determine *D*_r_ values precisely were not obtained under depolarized conditions for all the samples examined in this study.

**Figure 9:**
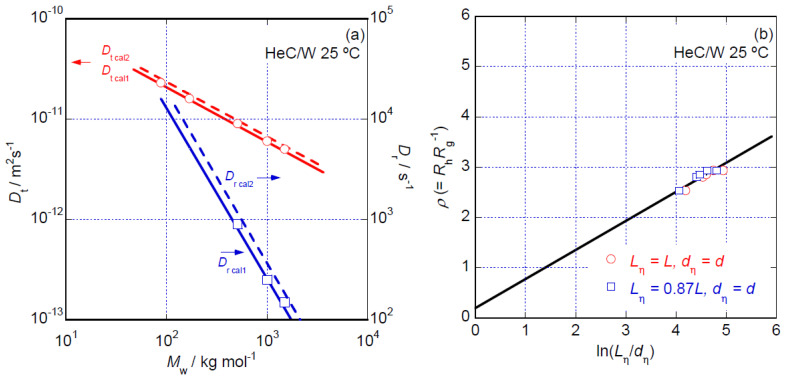
(**a**) *M*_w_ dependencies of *D*_t_ and *D*_r_ data for the HeC samples in aqueous solution at 25 °C. The solid lines show *D*_t cal1_ and *D*_r cal1_ calculated assuming *L*_h_ = *L* and *d*_h_ = *d*, and the broken lines show *D*_t cal2_ and *D*_r cal2_ calculated assuming *L*_h_ = 0.87*L* and *d*_h_ = *d,* as in the *M*_w_ dependencies of the [*η*] data seen in Figure 7. (**b**) Dependence of *ρ* (= *R*_h_*R*_g_^−1^) on ln(*L*_h_*d*_h_^−1^) for all the HeC samples in aqueous solution at 25 °C assuming the first condition, *L*_h_ = *L* and *d*_h_ = *d* (circular symbols), and the second condition, *L*_h_ = 0.87*L* and *d*_h_ = *d* (square symbols).

The *M*_w_ dependencies of the obtained *D*_t_ and *D*_r_ data for the HeC samples in aqueous solution are shown in Figure 9a. The *D*_t_ data for the HeC samples appear at a glance to demonstrate the relationship *D*_t_ ∝ *M*_w_^−0.65^, which is usually observed for flexible polymer chains in solution. However, because the SLS, S-WANS, S-WAXS and viscometric data strongly suggest that HeC molecules do not assume simple flexible polymer chain conformations and structures but rather appear as rigid rod-like structures in aqueous solution, we discuss the *D*_t_ and *D*_r_ data based on the rigid rod particle model [30]. According to Ortega and García de la Torre [30], *D*_r_ and *D*_t_ are theoretically expressed as

(2) $$D_{t\;\mathrm{cal}}=\frac{k_{B}T\left\{\ln(L_{\eta }/d_{\eta })+C_{t}\right\}}{3\pi \eta_{w}L_{\eta }},$$

(3) $$D_{r\;\mathrm{cal}}=\frac{3k_{B}T\left\{\ln(L_{\eta }/d_{\eta })+C_{r}\right\}}{\pi \eta_{w}L_{\eta }{}^{3}},$$

where *k*_BT_, *T*, *C*_t_ and *C*_r_ represent, respectively, the Boltzmann constant, the absolute temperature and numerical constants describing the contribution of hydrodynamic interactions for *D*_t_ and *D*_r_. The values of *C*_t_ and *C*_r_ are given as *L*_η_/*d*_η_ polynomials [30]. The solid lines shown in Figure 9a show the theoretical *D*_t cal1_ and *D*_r cal1_ calculated simply assuming *L*_h_ = *L* and *d*_h_ = *d*. The broken lines labeled *D*_t cal2_ and *D*_r cal2_ in Figure 9a were obtained assuming *L*_h_ = 0.87*L* and *d*_h_ = *d*, the same values that were used in the evaluation of the *M*_w_ dependency of the viscometric [*η*] data. The observed differences between *D*_t cal1_ and *D*_t cal2_ and between *D*_r cal1_ and *D*_r cal2_ are not significant. As the agreement between the obtained *D*_t_ data and *D*_t cal1_ (and *D*_t cal2_) and between the *D*_r_ data and *D*_r cal1_ (and *D*_r cal2_) is reasonable, we might conclude that the rigid rod particle model can explain the *M*_w_ dependency of the *D*_t_ and *D*_r_ data for the HeC samples examined in this study. Consequently, the existence of a rigid rod-like conformation and structure of HeC molecules in aqueous solution is strongly supported from the viewpoint of diffusional behavior.

The conformations and structures of solute molecules can be considered based on the value of the so-called shape factor (*ρ*) of the molecules. This factor is defined as the ratio of *R*_g_ to the hydrodynamic radius (*R*_h_) of the molecule given as *R*_h_ = *k*_B_*T*(6π*D*_t_)^−1^. If the particles formed by HeC molecules simply assume flexible coil-like conformations and structures in aqueous solution, the particles have a shape factor of *ρ* ~ 1.6 irrespective of the *M*_w_ of the particles [30]. The reason for this constant *ρ* value is that an identical *M*_w_ exponent is usually observed for both the *R*_g_ and *R*_h_ (∝ *D*_t_^−1^) data for many flexible polymer chain systems. On the other hand, if the particles of HeC molecules have a rigid rod-like conformation, the shape factor should satisfy the relationship *ρ* = 0.18 + 0.58ln(*L*_h_*d*_h_^−1^) for long particles [17,30] for which *L*_h_*d*_h_^−1^ > 10. Figure 9b shows the dependence of the *ρ* data on ln(*L*_h_*d*_h_^−1^) for all the HeC samples in aqueous solution evaluated using the simple first condition, *L*_h_ = *L* and *d*_h_ = *d*, and the second condition, *L*_h_ = 0.87*L* and *d*_h_ = *d*, as assumed in the discussion of viscometric behavior. Here, we must note that the relationship *L*_h_*d*_h_^−1^ > 10 is well satisfied for all the HeC sample solutions examined according to the SLS data analysis in which the rigid rod particle form factor above is assumed. The *ρ* data present inconstant values significantly greater than 1.6 and proportional to ln(*L*_h_*d*_h_^−1^); they approximately follow the theoretically predicted solid line, *ρ* = 0.18 + 0.58ln(*L*_h_*d*_h_^−1^), irrespective of the choice of conditions for *L*_h__,_ as shown in Figure 9b. These observations regarding the *ρ* data also strongly suggest that a rigid rod conformation and structure reasonably describe the formed particle structure of HeC molecules in aqueous solution.

We very recently reported that commercially available methyl cellulose, MC, samples also assume long rigid rod-like conformations and structures in aqueous solution irrespective of their *M*_w_ values, similar to the HeC samples in this study [30]. Therefore, it is likely that commercially available water-soluble chemically modified cellulose ethers such as HeC and MC samples have a tendency to assume rigid rod-like conformations and structures in aqueous solution and that this is an essential characteristic of these compounds that results from the strong physicochemical features of cellulose.

## 4. Conclusions

The conformation and structure of hydroxyethyl cellulose, HeC, ether samples with molar substitution numbers, *MS*, ranging from 2.36 to 2.41 and weight average molar masses, *M*_w_, ranging from 87 to 1.5 × 10^3^ kg mol^−1^, in aqueous solution were examined using the multiscattering techniques static and dynamic light scattering, neutron scattering and X-ray scattering and by viscometry. Although the form factors of flexible polymer chains and flexible cylinders cannot describe the obtained scattering vector, *q*, dependencies of the excess scattering intensity data obtained in the multiscattering measurements, the form factor of rigid rod particles of length *L* and diameter *d* dependent and the *M*_w_ of the molecular chains can reasonably explain the obtained scattering data. For HeC samples with *M*_w_ < 2 × 10^2^ kg mol^−1^, the rigid rod-like particle model predicts *L* values close to half of the contour length *l* and *d* ~ 1.4 nm. Thus, the formation of rigid rod-like particles resulting from a hairpin-like conformation and structure of HeC molecules is strongly suggested. The finding that the determined *L* of the formed particles is not proportional to *M*_w_ and that the ratio, *lL*^−1^, and the *d* values of the formed rigid rod-like particles increase with *M*_w_ for HeC samples with *M*_w_ > 2 × 10^2^ kg mol^−1^ indicates that the formed rod-like particles become thicker with increasing *M*_w__,_ maintaining their rigidity due to intramolecular attractive interactions such as hydrogen bond formation.

The translational and rotational diffusion coefficients determined using dynamic light scattering techniques and the intrinsic viscosities determined through viscometric measurements also strongly support the formation of rigid rod-like particles and an increase in rod diameter, *d*, with increasing *M*_w_.

## Figures and Tables

**Table 1:** Characteristics of the examined HeC samples: molar substitution number, *MS* (by hydroxyethyl groups), weight average molar mass, *M*_w_, radius of gyration, *R*_g_, second virial coefficient, *A*_2_, polydispersity index, *M*_w_*M*_n_^−1^, average molecular contour length, *l*, particle length, *L*, particle diameter, *d*, and ratios, *LR*_g_^−1^ and *lL*^−1^.

Code	*MS*	*M*_w_/kg mol^−1^	*R*_g_/nm	*A*_2_/mL mol g^−2^ *	*M*_w_ *M*_n_^−1^	*l*/nm	*L*/nm **	*d*/nm **	*LR*_g_^−1^	*lL* ^−1^
HeC87	2.38	87.0	27	1.0 × 10^−3^	2.0	161	93.0	1.4	3.4	1.7
HeC170	2.41	170	43	1.0 × 10^−3^	2.7	315	150	1.6	3.5	2.1
HeC520	2.36	520	80	4.9 × 10^−4^	4.0	972	290	2.5	3.6	3.4
HeC1000	2.39	1000	120	1.1 × 10^−3^	2.4	1860	420	3.0	3.5	4.4
HeC1500	2.36	1500	140	1.5 × 10^−3^	2.2	2800	500	5.0	3.6	5.6

* With uncertainty of ±2.0 × 10^−4^. ** Evaluated assuming rigid rod particles (see text).
